# Epidemiological features for primary lymphoma of the female genital tract patients and development of a nomogram to predict survival

**DOI:** 10.1080/07853890.2022.2046289

**Published:** 2022-04-13

**Authors:** Fei Peng, Jingwen Li, Shidai Mu, You Qin, Jiewen Ma, Lisha Ai, Yu Hu

**Affiliations:** a Institute of Hematology, Union Hospital, Tongji Medical College, Huazhong University of Science and Technology, Wuhan, China; b Cancer Center, Union Hospital, Tongji Medical College, Huazhong University of Science and Technology, Wuhan, China; c Collaborative Innovation Center of Hematology, Huazhong University of Science and Technology, Wuhan, China

**Keywords:** Primary lymphoma, female genital tract, SEER, incidence, prognosis, nomogram

## Abstract

**Background:**

Primary lymphoma of the female genital tract (PLFGT) is a sporadic extranodal lymphoma. Its epidemiology and prognosis are not fully recognized. Our study aimed to construct and validate prognostic nomograms for predicting survival for patients with PLFGT.

**Methods:**

Incidence rate from 1975 to 2017 and patients with PLFGT from 1975 to 2011 in the Surveillance, Epidemiology and End Results (SEER) database were retrospectively reviewed. The nomograms of overall survival (OS) and disease-specific survival (DSS) were established according to the multivariate Cox regression analyses. The concordance index (C-index) and calibration plots were used to demonstrate its robustness and accuracy.

**Results:**

A total of 617 PLFGT patients were identified. The overall incidence of PLFGT is 0.437/1,000,000 (adjusted to the US standard population in 2000) from 1975 to 2017. Age, histological subtype, Ann Arbor Stage, and therapeutic strategy were identified as independent prognostic factors for OS and DSS by multivariate Cox regression (*p* < .05). Nomograms to predict 1-, 5-, and 10-year OS and DSS were established. The C-index and calibration plots showed a good discriminative ability and an optimal accuracy of the nomograms. Patients were divided into three risk groups according to the model of OS.

**Conclusions:**

The incidence of PLFGT has increased in the past 40 years, and the nomograms were developed and validated as an individualized tool to predict OS and DSS for all PLFGT patients and DLBCL patients. All patients are divided into three risk groups to assist clinicians to identify patients at high-risk and choose the optimal individualized treatments for patients.

**Highlights**
- The incident of PLFGT and its subtypes were calculated and compared.
- Nomograms were constructed to predict the 1-, 5-, and 10-year OS and DSS.
- Patients are divided into the low-risk, medium-risk, and high-risk according total score of the nomogram.

## Introduction

Non-Hodgkin’s lymphoma (NHL) ranks seventh in terms of incidence among men and women, constituting 4% of new cancer cases and 3% of cancer-related fatalities annually [1]. Extranodal lymphoma accounts for approximately 25–40% of them [2]. But primary lymphoma of the female genital tract (PLFGT) is uncommon, accounting for 1.5% of extranodal NHL, mainly in the ovary [3–5].

The majority of PLFGT patients were middle-aged females, aged over 40 years [6,7]. Its clinical manifestation is not specific as vaginal bleeding, pelvic mass, vaginal secretion, and abdominal pain [5]. It is easy to be confused with the other malignant tumour at the genital tract [6,8]. Therefore, histological and immunophenotypic analyses for diagnosis are indispensable [9].

PLFGT has a good prognosis compared with other extranodal lymphoma. Ann Arbor stage was commonly applied to evaluate the outcome, and the international prognostic index (IPI) was developed to provide more accurate prediction of prognosis [10]. But there is no specified prognostic model of PLFGT for its low incidence, as most literature about PLFGT is single case reports.

Therefore, we conducted this research based on the Surveillance, Epidemiology, and End Results (SEER) database to explore epidemiological and clinical characteristics about PLFGT. Prognostic nomograms were established to assist clinicians in estimating the prognosis accurately.

## Material and methods

### Data source and patients enrollment

Information on patients with PLFGT was obtained from the SEER database by SEER Stat software, version 8.3.6, which contains cancer cases in 18 tumour registration centres and covers approximately 28% of the population in the United States. The annual incidence rate was extracted from 1975 to 2017 to study the trend of the incidence rate. All incidence rates are age-adjusted.

Patients data are extracted from 1975 to 2011 to follow-up at least 5 years. Lymphoma was identified by the International Classification of Diseases for Oncology Version 3 (ICD-O-3) histology codes 9590–9599, 9650–9729 and originated from the female genital tract was identified using the lesion number C50.1–C57.9.

Inclusion and exclusion criteria are established as followed to ensure the reliability of data. Inclusion criteria, (1) diagnosis by microscopically confirmed; (2) diagnosed between 1975 and 2011; (3) active follow-up. Exclusion criteria: (1) reporting from autopsy and date certificate; (2) unknown Ann Arbor stage.

Individual data derived from the SEER database included demographic data (age, race, year of diagnosis, marital status), tumour characteristics (primary site, histological subtype, Ann Arbor Stage), treatment strategy （surgery, radiation, chemotherapy）and survival information (survival months, vital status, cause of death).

Overall survival (OS) and disease-specific survival (DSS) are the endpoint of interest which are defined as the duration from the diagnosis of PLFGT to death or last follow-up due to any causes or PLFGT, respectively.

### Statistical analysis

The incident rates (age-adjusted to the standard population of the United States in 2000) were calculated by SEER stat. The Kaplan–Meier curves for OS and DSS were drawn and analysed by the log-rank test. All patients were randomly split into training and validation dataset at the ratio of 2:1. The hazard ratio (HR) and the associated 95% confidence interval (CI) were calculated by multiple cox regression analysis to identify independent risk factors, used to construct the nomograms. Internal and external validation were generated to measure the discrimination powers of the nomograms model using the concordance index (C-index) and calibration curve. In addition, patients were categorized into three different risk groups based on the total nomogram score of OS.

All statistical analyses were performed using R software (version 4.0.1) and X-tile (version 3.6.1). The R package included Table1, survival, survminer, rms, and ggplot2. A two-sided *p*-value < .05 was considered statistically significant.

## Results

### Incidence of PLFGT

**Figure 1.**
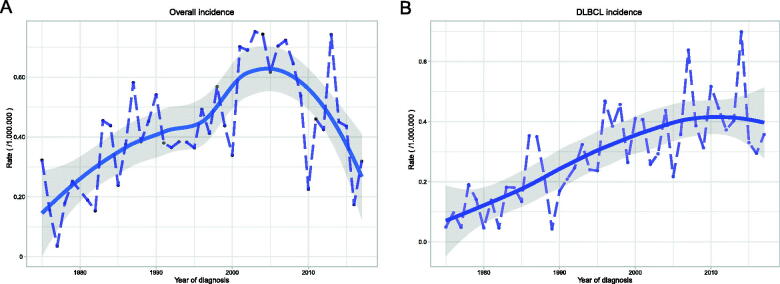
Incidence of PLFGT from 1975 to 2017 adjusted to the 2000 standard US: (A) All patients; (B) DLBCL patients.

The total incidence of PLFGT was 0.44/1,000,000 (adjusted to the US standard population in 2000) from 1975 to 2017. In the last 40 years, the incidence increased stably before 2005 and then decreased with incidence peaking from 1997 to 2007 for all types, but the incidence of DLBCL has been increasing (Figure 1). According to the race, the incidence of African-American (0.335/1,000,000) was lower than other people (0.410/1,000,000 for white, 0.439/1,000,000 for American Indians, Alaskan natives, and Asian/Pacific Islanders). Grouped by age, the incidence of patients upper 60 years was much higher than that of patients younger than 40 years and 40–59 years (Table 1).

**Table 1.** Incidence rate from 1975 to 2017.

	Rate (1,000,000)
Overall	0.437
Year of diagnosis	
1975–1985	0.196
1986–1996	0.403
1997–2007	0.586
2008–2017	0.398
Age	
＜40	0.150
40–59	0.594
≥60	1.005
Race	
White	0.410
African-American	0.335
American Indians, Alaskan natives, and Asian/Pacific Islanders	0.439
Pathological type	
DLBCL	0.271
FL	0.057
BL	0.026
MALT	0.022
SBL	0.006
TCL	0.004

### Demographics of PLFGT patients

A total of 617 eligible patients with PLFGT are identified according to inclusion and exclusion criteria. Patients are allocated to training cohort or validation cohort randomly based on the ratio of 2:1. The demographic and clinicopathological features are compiled in Table 2.

**Table 2.** Baseline demographic and clinical characteristics of patients.

	Overall	Training dataset	Validation dataset
	(*N* = 617)	(*N* = 411)	(*N* = 206)
**Year of diagnosis**			
1975–1984	17 (2.8%)	11 (2.7%)	6 (2.9%)
1985–1993	83 (13.5%)	59 (14.4%)	24 (11.7%)
1994–2002	191 (31.0%)	122 (29.7%)	69 (33.5%)
2003–2011	326 (52.8%)	219 (53.3%)	107 (51.9%)
**Age**			
Mean (SD)	55.3 (18.8)	55.0 (18.7)	55.9 (19.1)
Median [Min, Max]	55.0 [1.00, 94.0]	54.0 [7.00, 94.0]	55.0 [1.00, 93.0]
＜40	140 (22.7%)	97 (23.6%)	43 (20.9%)
41–50	135 (21.9%)	90 (21.9%)	45 (21.8%)
51–60	93 (15.1%)	59 (14.4%)	34 (16.5%)
61–70	82 (13.3%)	55 (13.4%)	27 (13.1%)
71–80	107 (17.3%)	74 (18.0%)	33 (16.0%)
≥80	60 (9.7%)	36 (8.8%)	24 (11.7%)
**Race**			
White	506 (82.0%)	340 (82.7%)	166 (80.6%)
African-American	56 (9.1%)	36 (8.8%)	20 (9.7%)
Asian or Pacific Islander	50 (8.1%)	31 (7.5%)	19 (9.2%)
American Indian/Alaska Native	5 (0.8%)	4 (1.0%)	1 (0.5%)
**Marital stutes**			
Married (including common law)	331 (53.6%)	220 (53.5%)	111 (53.9%)
Widowed/Separated	122 (19.8%)	74 (18.0%)	48 (23.3%)
Single (never married)	113 (18.3%)	83 (20.2%)	30 (14.6%)
Divorced	51 (8.3%)	34 (8.3%)	17 (8.3%)
**Primary Site**			
Ovary	239 (38.7%)	148 (36.0%)	91 (44.2%)
Cervix uteri	129 (20.9%)	95 (23.1%)	34 (16.5%)
Uterus	98 (15.9%)	62 (15.1%)	36 (17.5%)
Vagina	69 (11.2%)	47 (11.4%)	22 (10.7%)
Vulva	53 (8.6%)	37 (9.0%)	16 (7.8%)
others	29 (4.7%)	22 (5.4%)	7 (3.4%)
**Pathological type**			
DLBCL	380 (61.6%)	249 (60.6%)	131 (63.6%)
FL	80 (13.0%)	58 (14.1%)	22 (10.7%)
BL	36 (5.8%)	21 (5.1%)	15 (7.3%)
MALT	31 (5.0%)	21 (5.1%)	10 (4.9%)
SBL	8 (1.3%)	7 (1.7%)	1 (0.5%)
TCL	6 (1.0%)	4 (1.0%)	2 (1.0%)
others	76 (12.3%)	51 (12.4%)	25 (12.1%)
**Ann Arbor Stage**			
Stage I	278 (45.1%)	190 (46.2%)	88 (42.7%)
Stage II	107 (17.3%)	71 (17.3%)	36 (17.5%)
Stage III	35 (5.7%)	20 (4.9%)	15 (7.3%)
Stage IV	197 (31.9%)	130 (31.6%)	67 (32.5%)
**Surgery**			
No	214 (34.7%)	151 (36.7%)	63 (30.6%)
Yes	403 (65.3%)	260 (63.3%)	143 (69.4%)
**Radiation**			
No/Unknown	494 (80.1%)	325 (79.1%)	169 (82.0%)
Yes	123 (19.9%)	86 (20.9%)	37 (18.0%)
**Chemotherapy**			
No/Unknown	197 (31.9%)	133 (32.4%)	64 (31.1%)
Yes	420 (68.1%)	278 (67.6%)	142 (68.9%)
**Treatment modality**			
No treatment received	45 (7.3%)	31 (7.5%)	14 (6.8%)
Surgery only	120 (19.4%)	80 (19.5%)	40 (19.4%)
Radiotherapy only	15 (2.4%)	12 (2.9%)	3 (1.5%)
Chemotherapy only	101 (16.4%)	70 (17.0%)	31 (15.0%)
Radiotherapy + surgery	17 (2.8%)	10 (2.4%)	7 (3.4%)
Chemotherapy + surgery	228 (37.0%)	144 (35.0%)	84 (40.8%)
Chemotherapy + radiotherapy	53 (8.6%)	38 (9.2%)	15 (7.3%)
Chemotherapy + radiotherapy + surgery	38 (6.2%)	26 (6.3%)	12 (5.8%)

In the whole study cohort, the median and the mean age at diagnosis were 55.0 and 55.3 years. More than half of the patients (52.8%) were diagnosed between 2003 and 2011. Patients were more likely to be white (82.0%) and married (53.6%). The most common histopathological subtype of all patients was diffuse large B-cell lymphoma (DLBCL, 61.6%), followed by follicular lymphoma (FL, 13.0%), Burkitt lymphoma (BL, 5.8%), mucosa-associated lymphoma (MALT, 5.0%), small B lymphoma (SBL, 1.3%), and T cell lymphoma (TCL, 1.0%). The primary sites of most patients are in the ovary (38.7%) and cervix uteri (20.9%). According to Ann Arbor Stage, most patients were categorized as stage I (45.1%), followed by stage IV (31.9%), stage II (17.3%), and stage III (5.7%).

The overall diagnosis age is 55.3 years, but the age of diagnosis was lower in BL (33.9 years). And the mean overall survival of BL is the shortest among the B-cell lymphoma (Table 3). Among primary ovary lymphoma, 44.8% of patients are in stage IV of Ann Arbor Stage, but the median overall survival is the longest (121 months; Table 4). DLBCL was the most common histopathological subtype in all primary sites, but it was minor in primary vulva lymphoma (47.2%) (Table 5).

**Table 3.** Patient characteristics according to the histological subtypes.

	DLBCL	FL	BL	MALT	SBL	TCL
**No. of cases**	380	80	36	31	8	6
**Age,Mean (SD)**	55.6 (17.9)	56.3 (16.5)	33.9 (21.2)	60.7 (15.5)	72.1(21.8)	62.2(21.8)
**Race,White**	308 (81.1%)	67 (83.8%)	31 (86.1%)	24 (77.4%)	8(100.0%)	4 (66.7%)
**Marital status,Married**	206 (54.2%)	46 (57.5%)	12 (33.3%)	20 (64.5%)	4(50.0%)	3 (50.0%)
**Ann Arbor Stage, Stage IV**	121 (31.8%)	18 (22.5%)	21 (58.3%)	10 (32.3%)	2(25.0%)	2 (33.3%)
**Surgery performed**	233 (61.3%)	64 (80%)	31 (86.1%)	24 (77.4%)	6(75.0%)	2 (33.3%)
**Radiotherapy performed**	80 (21.1%)	16 (20.0%)	3 (8.3%)	6 (19.4%)	1(12.5%)	2 (33.3%)
**Chemotherapy performed**	289 (76.1%)	47 (58.8%)	30 (83.3%)	8 (25.8%)	1(12.5%)	3 (50.0%)
**Lymphoma as cause of death**	97 (25.5%)	14 (17.5%)	13 (36.1%)	1 (3.2%)	2 (25%)	3 (50.0%)
**Overall survival months**						
Mean (SD)	111 (94.4)	141 (84.8)	77.7 (76.9)	131 (63.1)	115 (50.1)	29.7 (53.9)
Median	99.5 [0, 403]	130 [4, 393]	35.0 [0, 251]	125 [15, 251]	101[79, 243]	7.5 [2,150]
1 year	309 (81.3%)	74 (92.5%)	22 (61.1%)	31 (100.0%)	8 (100.0%)	1 (16.7%)
5 year	250 (65.8%)	69 (86.3%)	18 (50.0%)	30 (96.8%)	8 (100.0%)	1 (16.7%)
10 year	158 (41.6%)	45 (56.3%)	13 (36.1%)	15 (48.4%)	1 (12.5%)	1 (16.7%)

**Table 4.** Patient characteristics according to the primary sites.

	Ovary	Cervix uteri	Uterus	Vagina	Vulva
**No. of cases**	239	129	98	69	53
**Age,Mean (SD)**	50.1 (19.5)	51.8 (15.9)	63.6 (15.5)	58.7 (19.2)	65.0 (19.6)
**Race,White**	206 (86.2%)	100 (77.5%)	78 (79.6%)	56 (81.2%)	43 (81.1%)
**Marital status,Married**	133 (55.6%)	75 (58.1%)	41 (41.8%)	41 (59.4%)	25 (47.2%)
**Ann Arbor Stage, Stage IV**	107 (44.8%)	30 (23.3%)	27 (27.6%)	11 (15.9%)	10 (18.9%)
**Surgery performed**	216 (90.4%)	66 (51.2%)	58 (59.2%)	14 (20.3%)	29 (54.7%)
**Radiotherapy performed**	16 (6.7%)	40 (31.0%)	20 (20.4%)	29 (42.0%)	12 (22.6%)
**Chemotherapy performed**	174 (72.8%)	91 (70.5%)	63 (64.3%)	52 (75.4%)	23 (43.4%)
**Lymphoma as cause of death**	65 (27.2%)	26 (20.2%)	28 (28.6%)	13 (18.8%)	12 (22.6%)
**Overall survival months**					
Mean (SD)	120 (97.2)	119 (88.2)	92.2 (76.3)	133 (107)	84.9 (75.6)
Median	121 [0, 401]	102 [0, 404]	83.5 [0, 327]	118 [1.00, 403]	73.0 [0, 310]
1 year	187 (78.2%)	111 (86.0%)	79 (80.6%)	61 (88.4%)	43 (81.1%)
5 year	161 (67.4%)	98 (76.0%)	63 (64.3%)	50 (72.5%)	31 (58.5%)
10 year	120 (50.2%)	57 (44.2%)	31 (31.6%)	34 (49.3%)	14 (26.4%)

**Table 5.** Percentage of different subtypes among the primary sites.

	Ovary	Cervix	Uterus	Vagina	Vulva
	239	129	98	69	53
**BL**	26 (10.9%)	5 (3.9%)	3 (3.1%)	0	1 (1.9%)
**DLBCL**	147 (61.5%)	81 (62.8%)	66 (67.3%)	46 (66.7%)	25 (47.2%)
**FL**	29 (12.1%)	23 (17.8%)	6 (6.1%)	6 (8.7%)	9 (17.0%)
**MALT**	5 (2.1%)	5 (3.9%)	10 (10.2%)	4 (5.8%)	4 (7.5%)
**others**	29 (12.1%)	14 (10.9%)	12 (12.2%)	9 (13.0%)	11 (20.8%)
**SBL**	3 (1.3%)	0	1 (1.0%)	2 (2.9%)	0
**TCL**	0	1 (0.8%)	0	2 (2.9%)	3 (5.7%)

### Survival analysis

**Figure 2.**
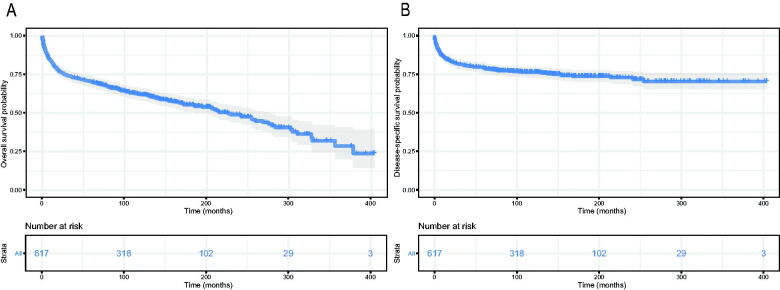
Survival analysis of PLFGT for all patients: (A) OS; (B) DSS.

**Figure 3.**
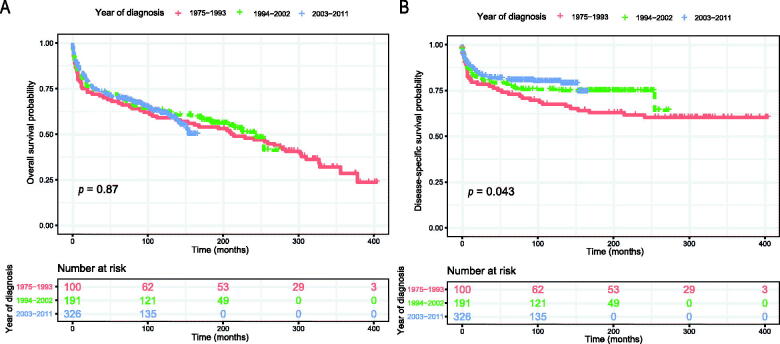
Survival analysis of PLFGT according to years of diagnosis: (A) OS; (B) DSS.

**Figure 4.**
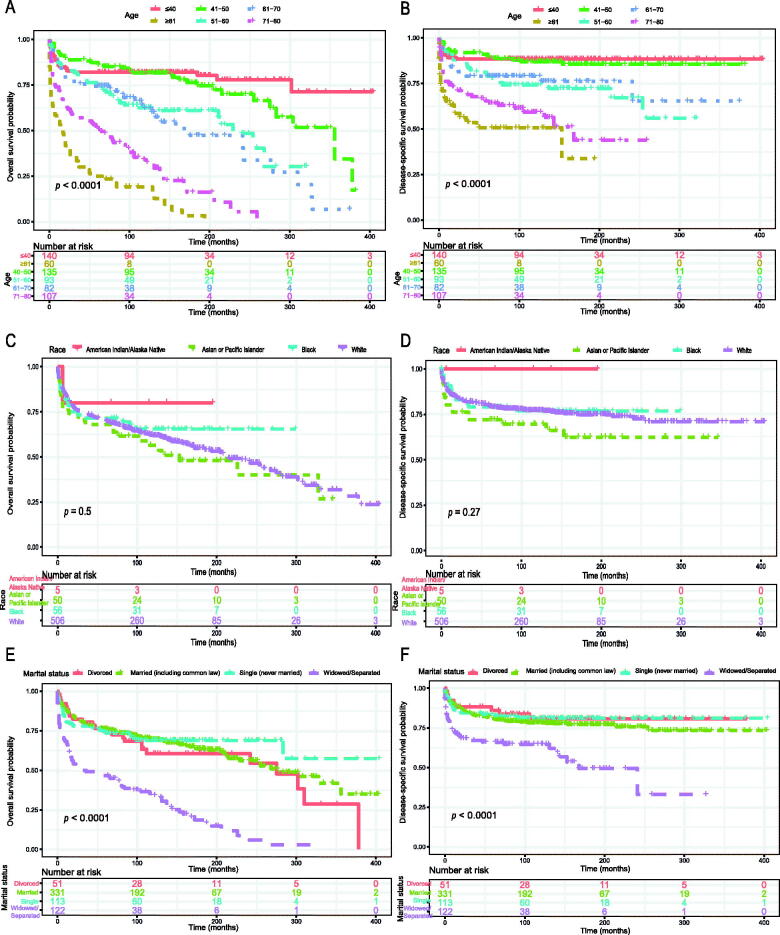
Overall survival of PLFGT according to (A) age, (C) race, and (E) marital status. Disease-specific survival of PLFGT according to (B) age, (D) race, and (F) marital status.

**Figure 5.**
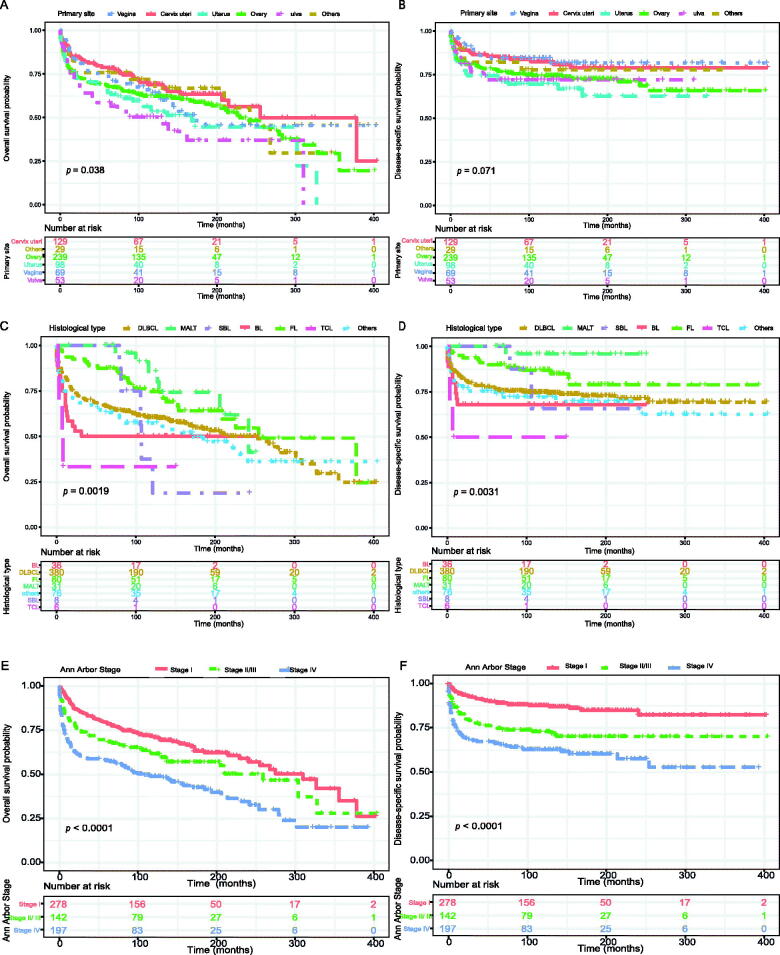
Overall survival of PLFGT according to (A) primary site, (C) histological type and (E) Ann Arbor Stage. Disease-specific survival of PLFGT according to (B) primary site, (D) histological type and (F) Ann Arbor Stage.

**Figure 6.**
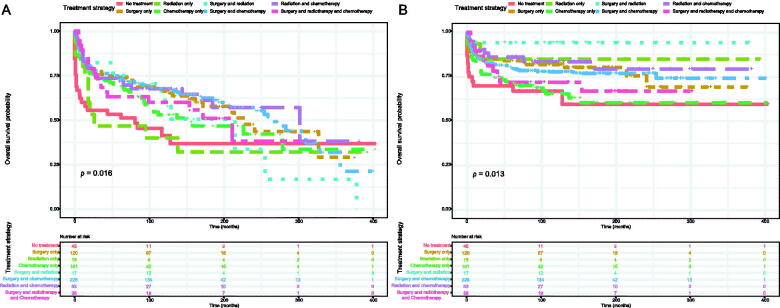
Survival analysis of PLFGT according to treatment strategy: (A) OS; (B) DSS.

The Kaplan–Meier method was used to evaluate the OS and DSS among all patients (Figure 2). The OS and DSS increase over time and decrease with age significantly. Patients younger and diagnosis later seem to have a better prognosis (Figures 3 and 4). Race has no impact on survival, but marital status has an impact on survival significantly. Patients widowed or separated had the shorter OS and DSS than others (Figure 4). According to tumour characteristics, pathological type and Ann Arbor Stage, rather than the primary site, were respectively related to the outcome of PLFGT patients. Stage IV patients with TCL had the worse OS and DSS significantly (Figure 5). The Kaplan–Meier curves for the treatment strategy are presented in Figure 6. OS and DSS improved significantly compared to untreated patients, but it was various by treatment strategy.

### Multivariable Cox regression analysis and nomogram

Multivariate Cox analysis was performed to identify the prognostic factors associated with the OS and DSS in patients and showing that age, histological type, Ann Arbor stage, and treatment strategy were independent prognostic factors (Table 6). Patients who received a combination of surgery and radiotherapy had the lowest HR for DSS of 0.071 (95% CI 0.009–0.560; *p* = .012), but it was not significant in OS.

**Table 6.** Multivariable Cox regression analysis of OS and DSS for all patients.

Variables	OS			DSS		
	HR	95% CI	*p* value	HR	95% CI	*p* value
**Years of diagnosis**						
1975–1984						
1985–1993	0.841	0.463–1.528	.569	0.636	0.322–1.256	.192
1994–2002	0.489	0.272–0.878	.017	0.399	0.207–0.771	.006
2003–2011	0.380	0.211–0.682	.001	0.223	0.115–0.432	<.001
**Age**						
≤40						
41–50	1.872	1.097–3.196	.022	1.595	0.766–3.321	.213
51–60	3.999	2.290–6.983	<.001	3.730	1.817–7.656	<.001
61–70	4.623	2.638–8.099	<.001	3.466	1.630–7.371	.001
71–80	10.911	6.372–18.682	<.001	8.034	3.966–16.275	<.001
	16.194	9.017–29.082	<.001	6.034	3.932–9.258	<.001
**Race**						
American Indian/Alaska Native						
Asian or Pacific Islander	1.321	0.170–10.248	.790	–	–	–
African-American	1.568	0.202–12.195	.667	–	–	–
White	1.432	0.193–10.633	.725	–	–	–
**Marital status**						
Divorced						
Married (including common law)	0.855	0.544–1.343	.496	1.451	0.714–2.949	.303
Widowed/Separated	1.153	0.696–1.912	.580	1.356	0.622–2.958	.444
Single (never married)	0.987	0.565–1.725	.963	1.277	0.553–2.947	.567
**Primary Site**						
Cervix uteri						
Ovary	1.185	0.799–1.757	.399	1.343	0.780–2.317	.288
Uterus	0.989	0.642–1.523	.960	1.557	0.875–2.769	.132
Vagina	0.757	0.463–1.238	.267	0.692	0.321–1.490	.347
Vulva	0.824	0.496–1.367	.453	0.854	0.404–1.804	.678
others	0.978	0.504–1.900	.948	1.209	0.478–3.055	.688
**Classification**						
BL						
DLBCL	0.346	0.200–0.596	<.001	0.331	0.163–0.674	.002
FL	0.188	0.097–0.364	<.001	0.167	0.067–0.412	<.001
MALT	0.109	0.043–0.275	<.001	0.036	0.004–0.290	<.001
SBL	0.169	0.057–0.500	.001	0.132	0.026–0.663	.014
TCL	1.003	0.296–3.405	.996	1.306	0.297–5.737	.724
Others	0.344	0.183–0.645	.001	0.342	0.150–0.784	.011
**Ann Arbor stage**						
Stage I						
Stage II/Stage III	1.295	0.918–1.828	.141	2.013	1.231–3.292	.005
Stage IV	2.284	1.681–3.102	<.001	3.720	2.381–5.811	<.001
**Treatment modality**						
No treatment received						
Surgery only	0.391	0.227–0.674	.001	0.365	0.176–0.755	.007
Radiotherapy only	0.705	0.323–1.539	.380	0.285	0.062–1.312	.107
Chemotherapy only	0.443	0.261–0.752	.003	0.451	0.226–0.898	.023
Radiotherapy + surgery	0.504	0.239–1.064	.072	0.071	0.009–0.560	.012
Chemotherapy + surgery	0.327	0.191–0.557	<.001	0.264	0.130–0.535	<.001
Chemotherapy + radiotherapy	0.388	0.210–0.718	.003	0.300	0.125–0.721	.007
Chemotherapy + radiotherapy + surgery	0.676	0.351–1.302	.242	0.610	0.260–1.430	.256

**Figure 7.**
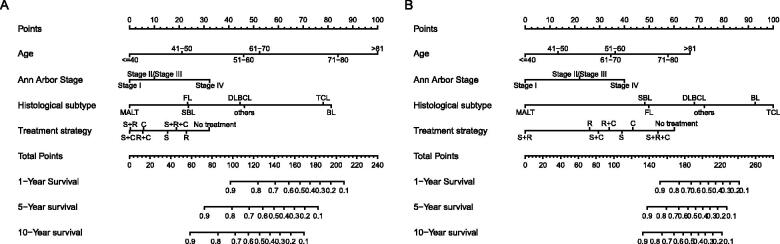
Nomograms to predict (A) overall survival and (B) disease-specific survival for patients with PLFGT.

**Figure 8.**
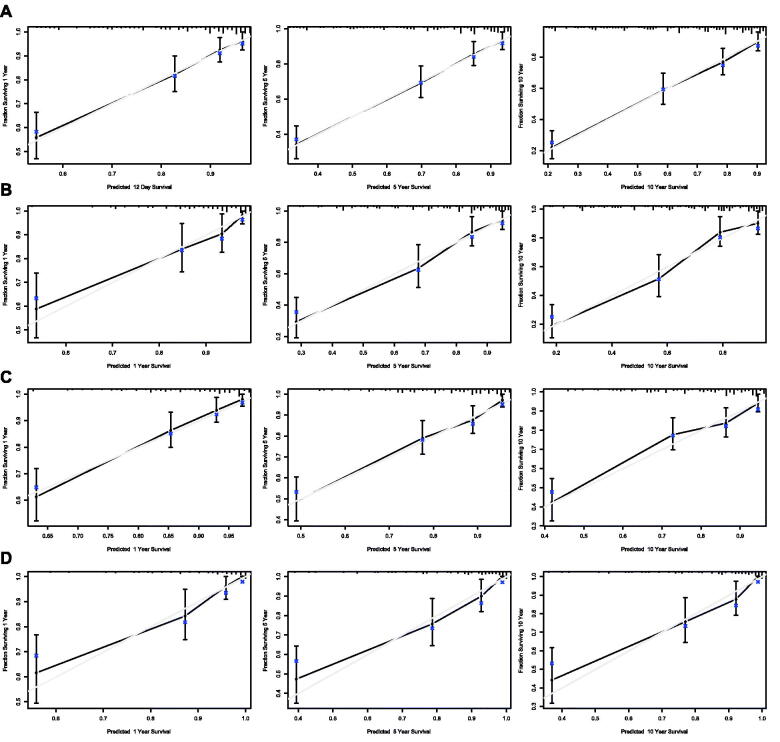
Calibration curves of the nomogram for 1-,5-, and 10-year overall survival of all patients in training set (A) and validation set (B), and calibration curves of the nomogram for 1-,5-, and 10-year disease-specific survival of all patients in training set (C) and validation set (D).

Nomograms for predicting 1-, 5- and 10-year OS and DSS were established based on the results of multivariate Cox analysis in the training dataset (Figure 7). The C-index for nomogram of OS was 0.759 (95% CI 0.731–0.788) in the training group and 0.789 (95% CI 0.754 − 0.825) in the validation group. For nomogram of DSS, the C-index was 0.752 (95% CI 0.717 − 0.788) in the training group and 0.823 (95% CI 0.782 − 0.866) in the validation group. The C-index indicates that all models were reliable. The calibration curves revealed high favourable consistency between the predicted and observed outcomes, indicating that the nomograms could be predictive accuracy (Figure 8).

**Figure 9.**
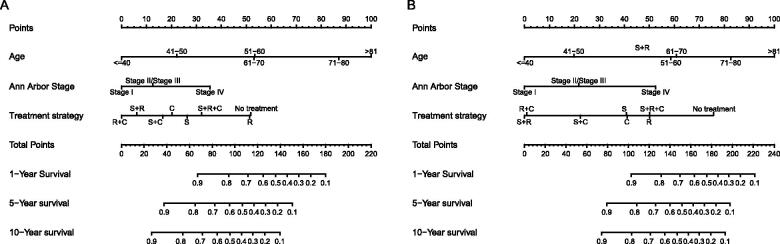
Nomograms to predict (A) overall survival and (B) disease-specific survival for patients with DLBCL.

**Figure 10.**
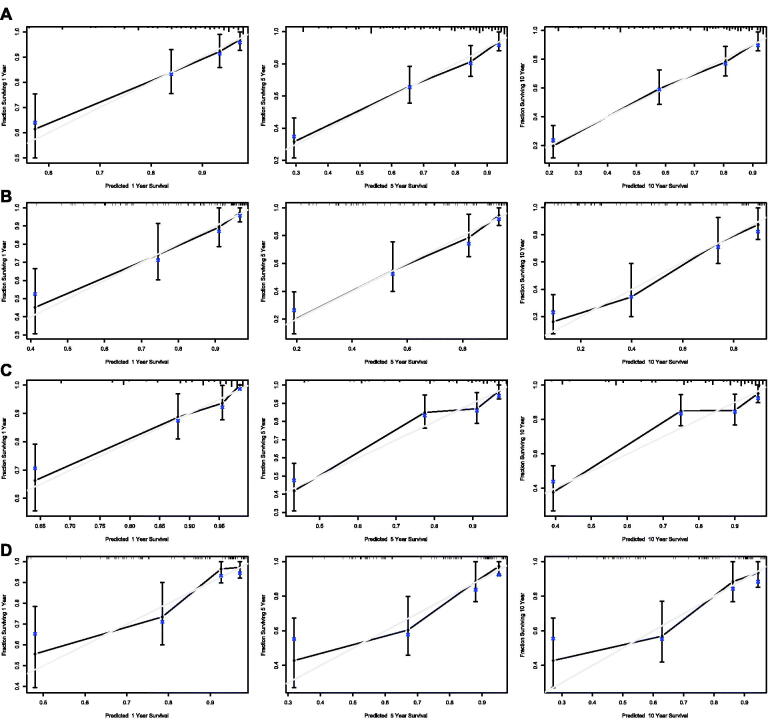
Calibration curves of the nomogram for 1-,5-, and 10-year overall survival of DLBCL patients in training set (A) and validation set (B), and calibration curves of the nomogram for 1-,5-, and 10-year disease-specific survival of DLBCL patients in training set (C) and validation set (D).

Further survival analysis was performed for DLBCL, the most common histopathological type lymphoma. The multiple cox analysis was shown in Table 7, and the nomograms for OS and DSS were established (Figure 9) The C-index for OS predictions in the training dataset and validation dataset were 0.783 (95% CI 0.756–0.811) and 0.726 (95% CI 0.685–0.770). The C-index for the prediction of DSS were 0.814 (95% CI 0.783–0.847) in training set and 0.741 (95% CI 0.706–0.778) in validation set, respectively. The calibration curves presented an excellent coherence between prediction and actual observation in both training cohort and testing cohort (Figure 10)

**Table 7.** Multivariable Cox regression analysis of OS and DSS for all patients.

Variables	OS			DSS		
	HR	95% CI	*p* Value	HR	95% CI	*p* value
**Years of diagnosis**						
1975–1993						
1994–2002	0.633	0.968–2.577	.067	1.431	0.762–2.686	.265
2003–2011	0.962	0.633–1.709	.877	0.704	0.364–1.362	.298
**Age**						
≤40						
41–50	1.895	0.937–3.834	.075	1.590	0.556–4.550	.387
51–60	4.517	2.231–9.148	.000	4.227	1.597–11.192	.004
61–70	3.997	1.945–8.211	.000	4.394	1.611–11.985	.004
71–80	10.173	5.033–20.561	.000	8.888	3.388–23.314	.000
	12.600	5.768–27.527	.000	13.310	4.629–38.285	.000
**Race**						
American Indian/Alaska Native						
Asian or Pacific Islander	1.414	0.174–11.471	.746	–	–	–
African-American	1.980	0.249–15.769	.519	–	–	–
White	1.504	0.200–11.323	.692	–	–	–
**Marital status**						
Divorced						
Married (including common law)	0.924	0.521–1.636	.785	1.787	0.695–4.591	.228
Widowed/Separated	1.465	0.769–2.791	.246	1.824	0.657–5.064	.249
Single (never married)	0.880	0.426–1.815	.729	1.083	0.344–3.409	.891
**Primary Site**						
Cervix uteri						
Ovary	1.513	0.873–2.623	.140	1.622	0.783–3.361	.193
Uterus	1.232	0.701–2.168	.469	1.748	0.849–3.596	.129
Vagina	0.794	0.409–1.541	.496	0.458	0.153–1.367	.161
Vulva	1.378	0.704–2.696	.349	1.307	0.517–3.305	.572
others	0.886	0.349–2.245	.798	1.323	0.409–4.279	.640
**Ann Arbor stage**						
Stage I						
Stage II/Stage III	1.347	0.856–2.120	.198	1.649	0.878–3.096	.120
Stage IV	2.400	1.601–3.598	.000	3.479	1.972–6.137	.000
**Treatment modality**						
No treatment received						
Surgery only	0.332	0.165–0.670	.002	0.309	0.127–0.751	.010
Radiotherapy only	0.785	0.286–2.151	.637	0.459	0.093–2.256	.338
Chemotherapy only	0.372	0.193–0.716	.003	0.404	0.178–0.916	.030
Radiotherapy + surgery	0.338	0.113–1.015	.053	0.081	0.010–0.685	.021
Chemotherapy + surgery	0.228	0.117–0.444	.000	0.177	0.076–0.413	.000
Chemotherapy + radiotherapy	0.230	0.104–0.508	.000	0.145	0.044–0.480	.002
Chemotherapy + radiotherapy + surgery	0.501	0.232–1.080	.078	0.438	0.163–1.175	.101

### Performance of the nomogram in stratifying risk

**Figure 11.**
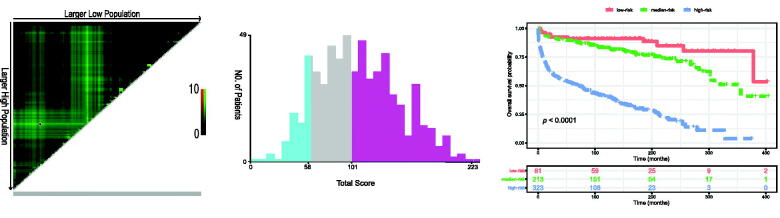
Cut-off values calculated by X-tile (A) and (B). Overall survival of all PLFGT patients stratified by risk (C).

To further verify the feasibility of our prediction model, all patients were stratified into low-, median-, and high-risk groups according to the nomogram-generated scores of OS. The cut-off values were 58 and 101, determined by X-tile software (Figure 11A and B). The Kaplan–Meier survival curves showed that high-risk patients (*n* = 323) significantly had the worst OS, and the low-risk patients (*n* = 81) had the best OS (*p* < .0001; Figure 11C)

## Discussion

PLFGT is extremely rare and most studies at present are case reports [11]. There is lacking prognostic analysis for PLFGT due to the low incidence and significant heterogeneity. Seer database is suitable for the study of PLFGT for its large sample size. So, we conducted this study based on the seer database to analyse the epidemiological trend and established nomograms to predict the prognosis of PLFGT.

Although PLFGT is exceptionally uncommon, our study showed that its incidence has increased in the last 40 years, especially in the period from 1997 to 2007. It is speculated that the causes of this increase include the rise of infectious factors such as the human immunodeficiency virus, the development of immunosuppressive therapy, the addition of the environmental exposure to pesticides and pollutants, and the improvements of the diagnostic techniques. [12]. Primary breast lymphoma also occurs in females commonly, but the prognosis of PLFGT is better than primary breast lymphoma [13]. Researchers suspect that hormonal stimulation could potentially influence the growth of PLFGT lesions as primary breast lymphoma [14]. But this has not been confirmed at present.

The clinical manifestations of PLFGT lack specificity [6]. “B symptoms” were uncommon at diagnosis compared with other lymphomas [15]. Some patients are even asymptomatic [16]. Therefore, lymphoma lesions are commonly misdiagnosed, causing the delay in diagnosis and reducing the therapeutic efficacy. Diagnostic imaging is essential for the correct diagnosis of pelvic masses suspected of gynecological lymphoma, but a definite diagnosis requires biopsy with histopathological evaluations and immunophenotyping [17]. Previous studies showed no lesions were detected in the diagnostic curettage for uterine lymphoma, and cervical lymphoma rarely invaded the mucosa, so deep-tissue aspiration biopsy is needed [18,19]. The prospective diagnosis of lymphoma avoids unnecessary surgery and enables the immediate institution of chemotherapy or radiation therapy [20,21].

The incidence of PLFGT rises with age in our research, which indicates a long‐term accumulated risk factors plays a vital role in the cause of PLFGT. The median age of overall patients was 55 years in our research, but various among the different primary sites and histopathological types [7]. Patients with the primary site in the ovary and cervix uteri tended to present younger than others in our cohort. Previous researches also suggest that lymphomas of uterine, vaginal, and vulvar tend to occur in elder women [22,23]. Primary uterine lymphomas occur in postmenopausal patients commonly but occasionally occur in women in their 20 s or 30 s [24,25]. But cervical lymphomas are inclined to present in premenopausal women [26]

Consistent with the most research, DLBCL was the most common type in the primary lymphoma of the female genital tract [27]. Farid Kosari et al. found that the incidence of DLBCL in the vulva was lower than lymphoplasmacytic lymphoma [7], which is conflicted with our study. The rate of DLBCL in the vulva was low relatively in our research, but it is still the largest proportion in all pathological types.

Similarly with other extranodal lymphoma, B-cell lymphomas are associated with better prognosis and overall response to treatment than TCL [15]. But it is worth noting that the BL patients have the lowest age of diagnosis and the worst prognosis among the patients of B-cell lymphomas. So, it is exceedingly imperative to distinguish BL [28].

The prognosis of PLFGT is excellent compared to other gynaecologic malignancies if diagnosis at early. DSS tends to increase with the year in our study for the alteration of treatment strategies, mainly targeted treatment, which has dramatically improved the prognosis of patients. But there is no recommended treatment strategy of PLFGT at present.

Our research showed that the radiotherapy and/or surgery can prolong DSS, but it is not conducive for prolonging OS. This maybe cause by the damage of surgery and radiotherapy to the organism, although surgery and radiotherapy could reduce the neoplastic mass and the risk of recurrence [29]. Surgery played a crucial role in the treatment many years ago [30]. But more conservative therapies are the mainstay treatment nowadays [10,19,31–33]. For young patients, chemotherapy alone was advocated in the earlier stage to preserve reproductive function [17,31,34]. Surgery was performed before NHL diagnosis at present [6]. The most frequently used first-line chemotherapy regimen for PLFGT was the CHOP regimen (cyclophosphamide, doxorubicin, vincristine, and prednisolone), which could prevent micrometastasis and preserve fertility [8]. Rituximab, an *anti-CD20* monoclonal antibody, is effective for the treatment of *CD20*-positive B lymphoma. The combination of rituximab and CHOP regimen (R-CHOP) further improved the survival of B-cell lymphoma [6,35].

The nomogram has been widely used as an essential prediction model to estimate individual survival [36]. But the nomogram for PLFGT patients is lacking for low incidence. Using the SEER database, our study constructed nomograms based on the age, histological type, and Ann Arbor Stage to provide a quantified survival prediction for individual PLFGT patients. The C-index and calibration plots showed excellent predictive performance of the nomograms.

According to the total score of the nomogram, patients were effectively divided into three groups (high-, middle- and low-risk groups) with the significant OS, which could assist clinicians in enabling personalized treatment.

However, the present study had several limitations. First, this stud is a retrospective study with inevitable inherent bias. Second, some potential independent prognostic variables, such as several biomarkers, B symptoms, and IPI, lacked in the SEER database. Third, the SEER database had no precise data on treatment. Thus, the therapeutic strategy was not included in the construction of nomograms. Therefore, high-quality studies with a larger sample size in future clinical work are indispensable. Despite these limitations, the SEER database remains a valuable resource in studying rare tumours for its large population. Our research still provided helpful information on the incidence, prognostic factors, and survival for PLFGT.

## Conclusion

The incidence of PLFGT has increased in the past 40 years. The present study established and validated nomograms that could accurately evaluate 1-, 5-, and 10- year OS and DSS for patients with PLFGT. This predictive models could assist clinicians to identify patients at high-risk and choose the optimal individualized treatments for patients.

## Data Availability

Publicly available datasets were analysed in this study. This data can be found in the SEER database (https://seer.cancer.gov/).

## Abbreviations

BL: Burkitt Lymphoma
C-index: concordance index
CI: confidence interval
DSS: disease-specific survival
DLBCL: diffuse large B-cell lymphoma
FL: follicular lymphoma
HR: hazard ratio
IPI: international prognostic index
MALT: mucosa-associated lymphoma
NHL: non-Hodgkin lymphoma
OS: overall survival
PLFGT: primary lymphoma of the female genital tract
SBL: small B lymphocytic
SEER database: Surveillance, Epidemiology, and End Results database
TCL: T cell lymphoma
